# Plasmazytoides Urothelkarzinom: eine seltene Ursache des lokalisierten Lymphödems

**DOI:** 10.1007/s00105-024-05358-z

**Published:** 2024-05-08

**Authors:** Sven-Niklas Burmann, Alena-Lioba Michalowitz, Frank Oellig, Alexander Kreuter, Johanna Matull

**Affiliations:** 1 Klinik für Dermatologie, Venerologie und Allergologie, Helios St. Elisabeth Klinik Oberhausen, Universität Witten/Herdecke, Josefstr. 3, 46045 Oberhausen, Deutschland; 2 Pathologie Mülheim an der Ruhr, Mülheim an der Ruhr, Deutschland; 3 Klinik für Dermatologie, Venerologie und Allergologie, Helios St. Johannes Klinik Duisburg, Universität Witten/Herdecke, Duisburg, Deutschland

**Keywords:** Plasmazytoides Urothelkarzinom, Harnblase, Histomorphologie, Lokalisiertes Lymphödem, Tumor-assoziiertes Lymphödem, Plasmacytoid urothelial carcinoma, Urinary bladder, Histomorphology, Localized lymphedema, Cancer-related lymphedema

## Abstract

Lokalisierte Lymphödeme der Genitalregion sind insgesamt seltene Erkrankungen. Liegt ihnen eine angeborene Fehlentwicklung des Lymphgefäßsystems zugrunde, werden sie als primäre Lymphödeme bezeichnet. Sekundäre Lymphödeme entstehen durch exogene Schädigung von Lymphgefäßen infolge von z. B. operativen Eingriffen, Übergewicht, Filariasis, Radiotherapie oder tumorösen Prozessen. Wir präsentieren den Fall eines Patienten mit lokalisiertem Lymphödem der Genitalregion, für das sich ein bisher nicht diagnostiziertes Urothelkarzinom als ursächlich erwies.

## Abstract (en)

Localized lymphedema of the genital region is a rare medical condition. It is named primary lymphedema if caused by a congenital malformation of the lymphatic system. Secondary lymphedemas might be induced by exogenous damage to lymphatic vessels as a result of surgical interventions, obesity, filariasis, radiotherapy or malignancy. We report a case of localized lymphedema of the genial region for which a previously unknown urothelial carcinoma turned out to be the underlying cause.

## Anamnese

**Abb. 1:**
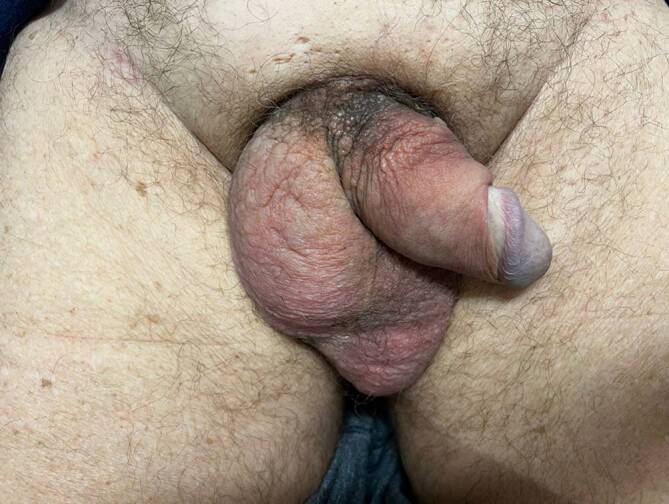
In der Übersichtsaufnahme zeigt sich ein großflächiges, derbes Ödem, welches Mons pubis, Peniswurzel und Skrotum einbezieht. Akzentuiert an der Radix penis zeigen sich multiple hautfarbene bis bräunliche, verruköse Papeln

Ein 79-jähriger Patient stellte sich mit einer binnen Wochen rasch progredienten Schwellung und Induration des Mons pubis, der Peniswurzel und des Skrotums in unserer dermatologischen Ambulanz vor (Abb. 1). Vorangegangen war den Hautveränderungen ein akutes Nierenversagen, welches in einer auswärtigen urologischen Klinik stationär therapiert worden war. Ein komplizierter Harnwegsinfekt war zu diesem Zeitpunkt die vermutete Ursache der Nierenfunktionsstörung. Passager erfolgte die Anlage eines transurethralen Blasenkatheters. Etwa eine Woche nach Entfernung des Katheters bemerkte unser Patient eine neue, rasch progrediente, schmerzhafte Schwellung der gesamten Genitalregion. Fieber oder Schüttelfrost bestanden zum Zeitpunkt der Vorstellung in unserer Klinik nicht.

## Hautbefund

**Abb. 2:**
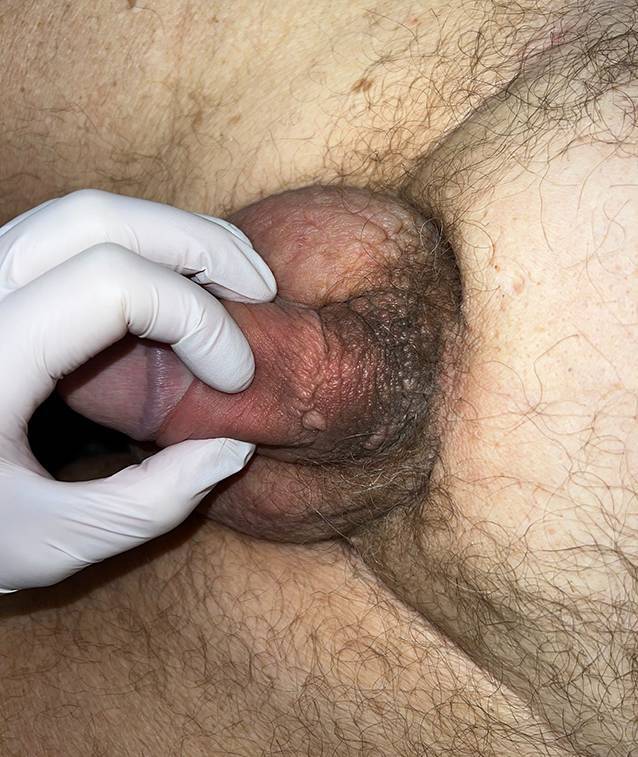
In der ventralen Nahaufnahme zeigen sich zirkulär im Bereich der Peniswurzel verruköse, hautfarbene bis bräunliche Papeln, die zu Plaques konfluieren. Auch in dieser Aufnahme imponiert ein gut abgrenzbares Skrotalödem

Im Bereich des Mons pubis, skrotal sowie an der Peniswurzel zeigte sich ein umschriebenes Ödem. Zudem imponierten verruköse, hautfarbene bis bräunliche, aggregierte, wenige Millimeter durchmessende Papeln an der Peniswurzel. Im Bereich des Mons pubis war die Haut Cellulite-ähnlich verändert („Orangenhaut“ oder „peau d’orange“) und induriert (Abb. 2). In dieser Lokalisation war zudem eine subkutane, nichtverschiebliche Raumforderung tastbar. Die lokoregionären Lymphknoten waren nicht vergrößert tastbar.

## Diagnostik

**Abb. 3:**
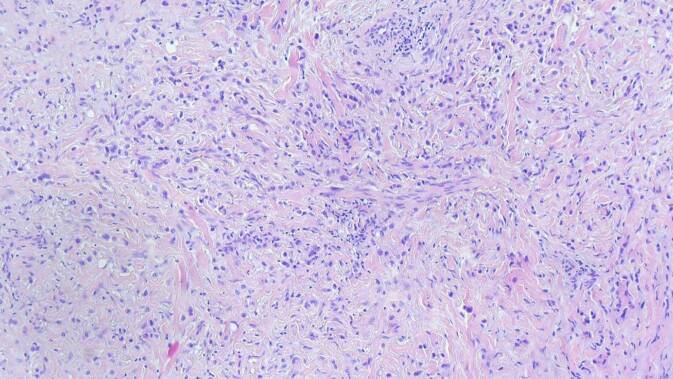
Diffus einzelzellig und infiltrativ wachsende, plasmazytoide Tumorzellen innerhalb der Lederhaut. (Hämatoxylin-Eosin-Färbung, Originalvergrößerung 100:1)

**Abb. 4:**
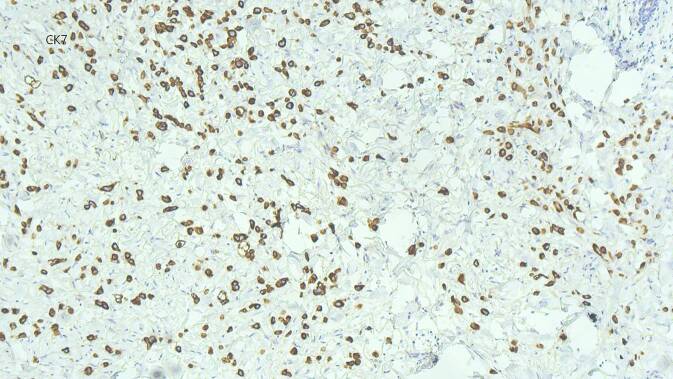
Immunhistochemische Untersuchung mit deutlicher Positivität für CK7. (Originalvergrößerung 100:1)

**Abb. 5:**
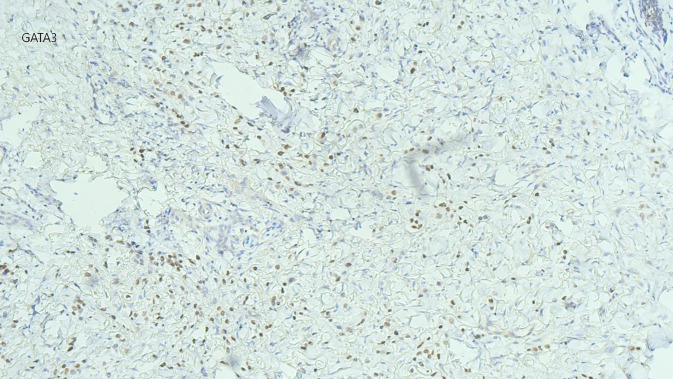
Immunhistochemische Untersuchung mit deutlicher Positivität für GATA3. (Originalvergrößerung 100:1)

Aufgrund der Akuität der Symptomatik, ungewöhnlichen Lokalisation des Ödems und raschen Progression entschieden wir uns zur Entnahme einer spindelförmigen Hautbiopsie zur weiterführenden Diagnostik. Histologisch zeigten sich im Bereich der Lederhaut diffus einzelzellig und infiltrativ wachsende, plasmazytoide Tumorzellen (Abb. 3). In den immunhistochemischen Zusatzuntersuchen zeigten diese Infiltrate CK20- und CK7-Reaktivität (Abb. 4), eine nukleäre Expression von GATA3 (Abb. 5) und teilweise Positivität gegenüber dem proliferationsassoziierten Antigen Ki67 (Mib1) bei negativer Reaktion gegenüber CK5/6, Östrogenrezeptor, CD68, Androgenrezeptor, CDX2 und NKX3.1.

## Labor

- Kreatinin 2,82 mg/dl (Normbereich: 0,5–1,1 mg/dl)
- Glomeruläre Filtrationsrate 20 ml/min (Normbereich: 90–120 ml/min)
- Hämoglobin 7,6 g/dl (Normbereich: 13,5–17,5 g/dl)
- Leukozyturie
- Geringgradige Proteinurie

## Diagnose

- Diffuse kutane Infiltration eines plasmazytoiden Urothelkarzinoms mit sekundärem, lokalisiertem Lymphödem

## Therapie und Verlauf

Klinik, Histologie und immunhistochemische Zusatzuntersuchungen sprachen für eine diffuse kutane Infiltration eines plasmazytoiden Urothelkarzinoms mit sekundärem, lokalisiertem Lymphödem. In einer ergänzenden MRT-Untersuchung des Abdomens und Beckens zeigte sich eine ausgeprägte, großflächige Wandverdickung der rechten lateralen, dorsalen und kranialen Anteile der Harnblase. Komplettierende Staginguntersuchungen konnten lokoregionäre Lymphknotenmetastasen und Fernmetastasen ausschließen. In transurethral gewonnenem Tumorgewebe ließ sich eine PD-L1(„programmed death ligand 1“)-Expression nachweisen. Aufgrund des lokal fortgeschrittenen Tumors mit diffuser Infiltration der Kutis (pT4b cN0 cM0, Stadium IVA nach WHO-Klassifikation 2016 [4]) erfolgte durch die behandelnden urologischen Kollegen schließlich die Einleitung einer Immuntherapie mit Pembrolizumab.

## Diskussion

Lokalisierte Lymphödeme der Genitalregion sind insgesamt seltene Erkrankungen. Von primären Formen sind sekundäre Formen abzugrenzen, die infolge einer exogenen Schädigung von Lymphgefäßen entstehen. Ihnen können Infektionen wie rezidivierende Erysipele oder urogenitale Tuberkulosen, operative Eingriffe, Radiotherapien oder – wie im geschilderten Fall – tumoröse Prozesse zugrunde liegen [2, 3, 6].

Differenzialdiagnostisch kann bei urogenital lokalisierten Lymphödemen auch ein sog. massives lokalisiertes Lymphödem („verrucous localized lymphedema“) vorliegen, welches Adipositas-assoziiert auftritt und sich mit polypoiden Läsionen der Inguinalregion und/oder der unteren Extremität manifestiert. Vermutete Ursache dieser Erkrankung ist eine Kompression lokaler Lymphgefäße durch schwere, herabhängende Körperfalten [8].

Unabdingbar ist der Ausschluss maligner Grunderkrankungen. Eine Infiltration der Haut durch Urothelkarzinome wie im vorliegenden Fall ist jedoch selten. Deutlich häufiger tritt eine kutane Beteiligung bei Mammakarzinomen auf [5]. Eine Infiltration von Lymphgefäßen durch Tumorzellen (Lymphangiosis carcinomatosa) führt zu „peau d’orange“-artigen Veränderungen der Hautoberfläche. Ein weiterer Mechanismus in der Entstehung maligner Lymphödeme ist eine direkte, tumorbedingte Kompression lokoregionärer Lymphgefäße. Meist führt eine Kombination beider Mechanismen zur Ausbildung erheblicher ödematöser Schwellungen [9].

Insbesondere bei umschriebenen Lymphödemen ist daher eine sorgfältige klinische Untersuchung der betroffenen Region inklusive Palpation der angrenzenden Lymphknotenstationen vorzunehmen. Ein noch unentdeckter Tumor – wie in unserem Fall –, aber auch ein Rezidiv eines bereits bekannten Tumors sollten ausgeschlossen werden. Ergänzende radiologische Diagnostik kann zur weiteren Einordnung hilfreich sein. Ein akuter Beginn der Symptomatik, rasche Progression, Schmerzhaftigkeit und tastbare Tumorinfiltrationen sind wichtige Kriterien zur Unterscheidung maligner Lymphödeme von benignen Formen [9].

Das bei unserem Patienten diagnostizierte plasmazytoide Urothelkarzinom gehört zu den High-grade-Tumoren mit aggressivem Verlauf und schlechter Prognose [4]. Zur Therapie lokal fortgeschrittener oder nichtresektabler Tumoren werden neben Cisplatin-basierten Chemotherapien zunehmend Checkpointinhibitoren eingesetzt. Insbesondere der PD-1(„programmed cell death protein 1“)-Antikörper Pembrolizumab gehört mittlerweile zu den etablierten Therapieverfahren für Karzinome mit positivem PD-L1-Status [7]. Analog zum malignen Melanom wird eine Therapie mit Checkpointinhibitoren auch im neoadjuvanten Setting beim muskelinvasiven Harnblasenkarzinom diskutiert und scheint nach aktuellem Kenntnisstand vorteilhaft hinsichtlich des progressionsfreien Überlebens zu sein [1].

## Fazit für die Praxis

- Lokalisierte Lymphödeme der Urogenitalregion bedürfen einer weiteren diagnostischen Abklärung.
- Als sog. sekundäre maligne Lymphödeme können sie einziges Symptom eines klinisch noch unentdeckten Tumors sein.
- Insbesondere akuter Beginn, rasche Progression und Schmerzhaftigkeit sind Hinweise für das Vorliegen eines malignen Lymphödems und sollten eine dezidierte Diagnostik nach sich ziehen.
